# Development and Validation of an Ultra-High Performance Liquid Chromatography-Tandem Mass Spectrometry Method for Simultaneous Determination of Four Type B Trichothecenes and Masked Deoxynivalenol in Various Feed Products

**DOI:** 10.3390/molecules21060747

**Published:** 2016-06-08

**Authors:** Zhichen Fan, Bing Bai, Peng Jin, Kai Fan, Wenbo Guo, Zhihui Zhao, Zheng Han

**Affiliations:** 1 Institute for Agro-food Standards and Testing Technology, Shanghai Academy of Agricultural Sciences, Shanghai 201403, China; zhichen0711@outlook.com (Z.F.); whitebing2@hotmail.com (B.B.); jinpeng_328@163.com (P.J.); Fankai1983@gmail.com (K.F.); guo1103bo@126.com (W.G.); zhao9912@hotmail.com (Z.Z.); 2 College of Food Science & Technology, Shanghai Ocean University, Shanghai 201306, China

**Keywords:** type B trichothecenes, masked deoxynivalenol, HLB cartridges, feed products, ultra-high performance liquid chromatography-tandem mass spectrometry

## Abstract

A reliable and sensitive analytical method was developed for simultaneous determination of deoxynivalenol(DON), 3-acetyldeoxynivalenol (3-ADON), 15-acetyldeoxynivalenol (15-ADON), fusarenon X (FUS-X), and masked deoxynivalenol (deoxynivalenol-3-glucoside, D3G) in formula feed, concentrated feed, and premixed feed products. The method was based on an improved sample pretreatment with the commercially available HLB cartridges used for sample purification and enrichment followed by analysis using ultra-high performance liquid chromatography-tandem mass spectrometry (UHPLC-MS/MS). Several key parameters including the extraction solvents, the positions of sample loading solvents, washing and elution solvents for HLB cartridges were carefully optimized to achieve optimal extraction and purification efficiencies. The established method was extensively validated by determining the linearity (R^2^ ≥ 0.99), sensitivity (limit of quantification in the range of 0.08–4.85 μg/kg), recovery (79.3%–108.1%), precision (Intra-day RSDs ≤ 13.5% and Inter-day RSDs ≤ 14.9%), and then was successfully applied to determine the four type B trichothecenes and D3G in a total of 31 feed samples. Among them, 26 were contaminated with various mycotoxins at the levels of 2.1–864.5 μg/kg, and D3G has also been detected in 17 samples with the concentrations in the range of 2.1–34.8 μg/kg, proving the established method to be a valuable tool for type B trichothecenes and masked DON monitoring in complex feed matrices.

## 1. Introduction

Type B trichothecenes, mainly including deoxynivalenol (DON), 3-acetyldeoxynivalenol (3-ADON), 15-acetyldeoxynivalenol (15-ADON), and fusarenon X (FUS-X), are widespread mycotoxins, which can be produced by *Fusarium* species, *i.e.*, *F. graminearum* and *F. culmorum* [1,2]. DON is one of the most abundant contaminants in animal feed products worldwide, and the most frequently occurring mycotoxin in China [3,4,5,6,7]. Acute and chronic ingestion of DON by animals can cause a variety of toxic effects, including feed refusal, weight loss, and vomiting [8]. 3-ADON and 15-ADON are the acetylation products of DON, with the hydroxyl at the C-3 or C-15 position being acetylated, which could also be produced directly by fungi. These two mycotoxins frequently co-occur with DON, and reportedly, more than 30% of the DON-contaminated samples contained one or two derivatives [9,10,11]. Equivalent or even stronger toxicity of 3-ADON and 15-ADON compared to that of DON has been elicited by hydrolyzing these conjugates into their toxic parent forms during mammalian digestion [12]. FUS-X is also frequently observed along with DON. Although scarce toxicity data could be found, it has been reported to induce adverse health effects, especially apoptosis in mice [13]. The contamination of type B trichothecenes in various feed products has led to serious economic losses and even posed high potential health risks to animal and humans in China and even all over the world [14].

Recently, some modified mycotoxins especially the glucoside and sulfate conjugates of the parent mycotoxins have been found, and draw more and more attention around the world [15,16]. These mycotoxins could not be detected by conventional analytical techniques because they are formed as detoxification metabolites with the structures changed in the plant, and thus are designated masked mycotoxins. Among all masked mycotoxins, deoxynivalenol-3-glucoside (D3G) is the most important one, which has been frequently detected in animal feed, maize, and wheat [15,17,18,19], with the concentration levels even higher than 1000 μg/kg. The contamination of D3G in feeds and grains has become a worldwide issue due to the surveys from the US [20], Austria [21], China [15], and the Czech Republic [22]. D3G has been reported to be cleaved by intestinal bacteria and become bioavailable as DON, and thus contribute to the total dietary exposure to DON, resulting in additional risks to animal and human health [23,24]. Therefore, it is an essential issue to develop a reliable analytical method for simultaneous determination of type B trichothecenes and D3G in animal feed products.

Various analytical methods based on thin-layer chromatography (TLC), enzyme-linked immune-sorbent assay (ELISA), and high-performance liquid chromatography (HPLC) have been established for determination of type B trichothecenes in animal feed products [25,26,27]. Poor separation, low sensitivity, and unsatisfying accuracy limit the application of TLC. ELISA methods were used for screening purposes and the positive results should be confirmed by other analytical techniques. HPLC combined with different detectors have been utilized as highly selective and sensitive methods for determination of mycotoxins, but conventional HPLC approach often costs a lot of time to separate the targeted analytes. These problems have been successfully solved by introducing ultra-HPLC (UHPLC) resulting in shorter run time, better chromatographic resolution, and improved sensitivity. The availability of ionization sources, *i.e.*, electrospray (ESI) and atmospheric pressure chemical ionization (APCI), has promoted the UHPLC tandem mass spectrometry (UHPLC-MS/MS) to be the most promising approach for multiple mycotoxins analysis. However, the complex interferences from the animal feed samples can significantly interfere in the separation and ionization process leading to the inaccuracy of the results and contaminating the expensive equipment, as a consequence, an efficient clean-up method is necessary for a reliable UHPLC-MS/MS method. In previous studies, liquid-liquid extraction (LLE), dispersive solid-phase extraction (DSPE), and solid phase extraction (SPE) have been used for sample pretreatment [15]. LLE is simple with the disadvantages of being time- and solvent-consuming. DSPE can be used for clean-up of these mycotoxins, but the procedure is also solvent-consuming and the purification efficiency is frequently not satisfactory with relatively high matrix effects and low sensitivity observed. SPE is being increasingly used due to its high enrichment factor, less solvent consumption and efficient elimination of interferences. However, as reported, until now, HLB SPE cartridges with different adsorbents can only be used for purification of type B trichothecenes (recovery of 80%–120%), but not suitable for D3G clean-up (recovery of 50%–70%) [15,18].

Therefore, the purpose of the present study was to develop a reliable UHPLC-MS/MS method for simultaneous determination of the four type B trichothecenes and masked DON in feedstuff based on an improved sample pretreatment using commercially available HLB cartridges for sample purification and enrichment. The established method was extensively validated by determining linearity, sensitivity, recovery, precision, and further applied for simultaneous determination of type B trichothecenes and masked DON in formula feed, concentrated feed, and premixed feed products to reveal the real contamination levels in Shanghai, China.

## 2. Results and Discussion

### 2.1. Optimization of Sample Preparation

#### 2.1.1. Selection of Extraction Method

**Figure 1:**
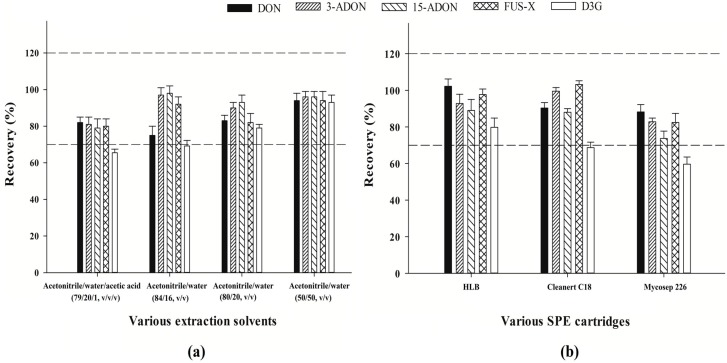
Comparison of the extraction efficiencies of the five mycotoxins by the four candidate extraction solvents (**a**) and purification efficiencies by three different clean-up cartridges (**b**) using blank formula feed samples spiked with 50 μg/kg of each mycotoxin. The error bar represents standard deviation (*n* = 3) and acceptable recoveries are in the range of two dashed lines (70%–120%).

Four frequently used extraction solvents including (i) acetonitrile/water/acetic acid (79/20/1, *v*/*v*/*v*); (ii) acetonitrile/water (84/16, *v*/*v*); (iii) acetonitrile/water (80/20, *v*/*v*); and (iv) acetonitrile/water (50/50, *v*/*v*) were compared in the present study by using the blank formula feed samples spiked with 50 μg/kg of each analyte. The recoveries were calculated by comparing the concentrations of the targeted analytes spiked before extraction to those after extraction. As shown in Figure 1a, when solvent (i) or solvent (ii) was utilized, although the recoveries of DON, 3-ADON, 15-ADON, and FUS-X were more than 70%, the recovery of D3G was poor (only 65.1% for solvent (i) and 69.2% for solvent (ii)). Satisfactory recoveries were obtained by using solvent (iii) or solvent (iv). Alternatively, solvent (iv) (acetonitrile/water, 50/50, *v*/*v*) was finally chosen as the extraction solvent due to its relatively high recovery rates for all mycotoxins.

#### 2.1.2. Optimization of SPE Clean-Up Procedure

For optimization of SPE clean-up procedure, the blank formula feed samples were also utilized. The samples were extracted as described in Section 3.4. The extraction solutions were collected, and used for comparison of purification efficiencies by three different clean-up cartridges, *i.e.*, *GPD* HLB, Cleanert C18 and Mycosep 226 cartridges. The recoveries were calculated by comparing the concentrations of the targeted analytes spiked before clean-up steps to those after clean-up procedures (The spiked concentration level was 50 μg/kg for each analyte). As shown in Figure 1b, the recovery values of DON, 3-ADON, 15-ADON, and FUS-X were all higher than 70% in any case, but for D3G, only when *GPD* HLB cartridge was used, the recovery rate could achieve more than 70%. Therefore, *GPD* HLB cartridge was selected.

**Figure 2:**
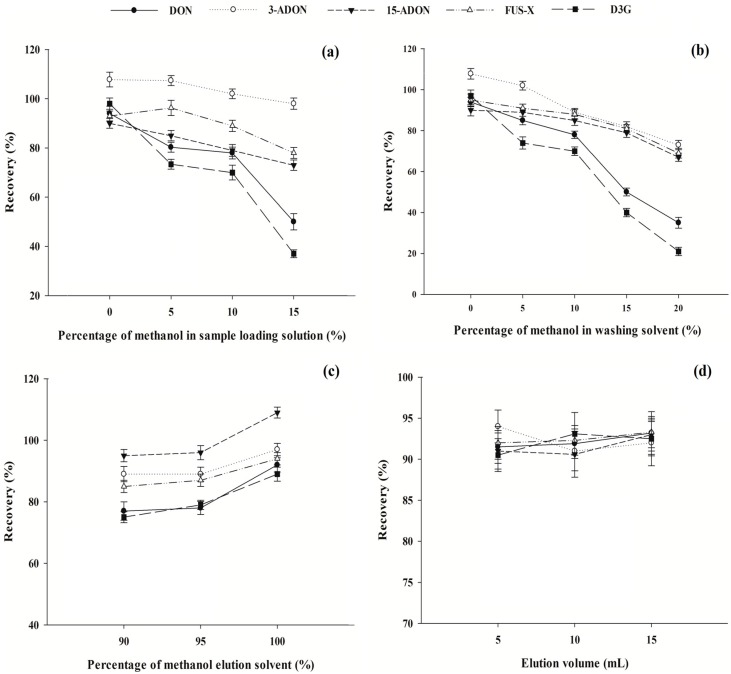
Effects of key parameters on the performance of HLB cartridges including the percentage of methanol in sample loading solution (**a**); the percentage of methanol in washing solvent (**b**); the percentage of methanol in elution solvent (**c**), and the elution volume (**d**). The concentrations of mycotoxins tested were 50 μg/kg (*n* = 3).

After selection of the SPE cartridge, the clean-up process was further optimized including the sample loading solution, and the washing and elution solvent. Different percentages of methanol (0%, 5%, 10% and 15%, *v*/*v*) in sample loading solutions were compared. The results showed that all analytes could be adsorbed in SPE cartridges only when pure water was used. Even when 5% methanol was added, the recovery rates were significantly decreased especially for DON and D3G with the values dropping from 94.1% to 80.3% and from 98.1% to 74.5%, respectively (Figure 2a). This might be used at least in partial for explanation why in the previous studies HLB cartridges were only used for clean-up of type B trichothecenes but not for D3G [15,28]. Since DON, 3-ADON, 15-ADON, FUS-X, and D3G were all water-soluble, the extraction solvent should be dried first to remove acetonitrile and then re-dissolved in pure water to prepare sample loading solution. As expected, only when water was selected as the washing solvent, satisfactory recovery rates could be obtained with the values in the range of 90.1%–107.8% (Figure 2b). Generally, the percentage of methanol in the elution solvent has a significant effect on the purification efficiency of the *GPD* HLB cartridges. Three different elution solvents with the percentages of methanol of 90%, 95%, and 100% (*v*/*v*) were compared. As shown in Figure 2c, the recoveries of all mycotoxins were obviously improved along with the increasing percentages of methanol. Satisfactory recoveries with the values from 89.1% to 99.8% for different mycotoxins were obtained when 100% methanol was selected as the elution solvents. To make the clean-up procedure more effective and economical, different amounts of elution solvents (5, 10, and 15 mL) were tested. The results are presented in Figure 2d. The use of 5 mL of methanol has already resulted in recovery rates of 90.5%–94.0%, while the use of higher amounts of methanol could not obviously increase the recovery; as a consequence, 5 mL of methanol were used.

#### 2.1.3. Evaluation of the HLB SPE Clean-up Method

**Figure 3:**
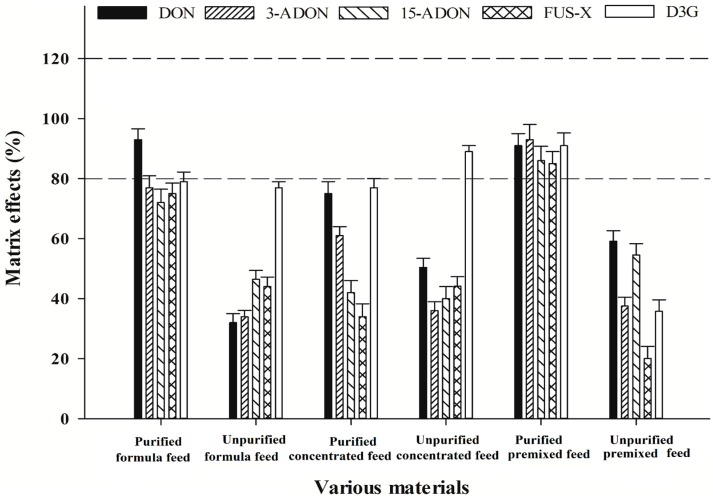
The matrix effectsof the five targeted mycotoxins in different feed matrices purified or not purified by HLB cartridges (*n* = 3). The spiked concentration level is 50 μg/kg for each mycotoxin. The tolerance level of matrix effects is in the range of the two dashed lines (80%–120%).

To characterize and clarify the established clean-up procedure, the matrix effects of the five mycotoxins in different feed products purified or not purified with HLB cartridges were compared. As shown in Figure 3, if the samples were not purified by HLB cartridges, the matrix effects were in the range of 13.0%–77.2% for formula feed, 34.4%–89.0% for concentrated feed, and 16.9%–59.1% for premixed feed, respectively. After clean-up, the matrix effects of all mycotoxins were efficiently eliminated with acceptable values in the range of 72.0%–93.2% and 85.8%–93.1% for formula feed matrix and premixed feed matrix, respectively. With regard to concentrated feed, although the matrix effects have been reduced by using the clean-up method, unsatisfactory values in the range of 34.3%–77.5% were obtained in concentrated feed sample. Therefore, matrix-matched calibration curves were used for quantification, in which, the concentrations of targeted mycotoxins in the feed sample were calculated using the calibrations constructed in the same feed matrix, so as to eliminate the matrix effects to ensure the accuracy of the established method.

### 2.2. Optimization of UHPLC-MS/MS Conditions

The ACQUITY UPLC^®^HSS T3 (2.1 × 150 mm, 1.8 μm; Waters, Milford, MA, USA) column was selected for separation of the targeted analytes by comparing different mobile phases to achieve an optimal separation and ionization efficiency for targeted mycotoxins. First, two frequently used mobile phases including methanol-water and acetonitrile-water were compared. The results showed that the separation and ionization efficiencies of all analytes were obviously higher when methanol was selected compared to acetonitrile. Then, different additives, *i.e.*, 1 mmol/L ammonium acetate, 5 mmol/L ammonium acetate, and 0.1% formic acid, were investigated. The results showed that when 5 mmol/L ammonium acetate or 0.1% formic acid was added, the ionization efficiency of all mycotoxins was obviously suppressed, and consequently, the responses of all mycotoxins were significantly decreased. Although the mobile phase containing 1 mmol/L ammonium acetate or not lead to almost the same responses for all mycotoxins, 1 mmol/L ammonium acetate was selected in the present study, due to its better separation efficiencies for 3-ADON and 15-ADON. Therefore, methanol-water containing 1 mmol/L ammonium acetate was finally selected as the mobile phase. Under such a situation, symmetrical peaks with identical half-peak width were obtained for each analyte and the retention times were 3.1, 5.6, 5.5, 3.8, and 2.8 min for DON, 3-ADON, 15-ADON, FUS-X, and D3G, respectively.

### 2.3. Method Validation

The established method was carefully validated by determining the following parameters: linearity, sensitivity, recovery, and precision in formula feed, concentrated feed and premixed feed products, respectively.

As shown in Table 1, nice linear relationships for all targeted mycotoxins were obtained both in neat solvent and in different feed matrices with the coefficients of determination (*R*^2^) more than 0.99. The limit of detection (LOD) and limit of quantification(LOQ) values for the five mycotoxins were in the range of 0.08–2.31 μg/kg and 0.10–4.85 μg/kg, respectively, proving that the sensitivity of the established method was much higher than the values reported in the previous studies, especially for D3G [15,18,29,30]. All targeted mycotoxins analyzed by the established method shown satisfactory recoveries, with the mean values in the range of 85.7%–102.3% for formula feed, 78.0%–101.8% for concentrated feed, and 78.5%–108.1% for premixed feed, respectively (Table 2). The intra- and inter-RSD values were in the range of 1.1%–13.5% and 2.6%–14.9%, respectively. All these validation results indicated that the developed analytical method was sensitive, accurate, repeatable, and could be utilized for simultaneous determination of DON, 3-ADON, 15-ADON, FUS-X, and D3G in different animal feed products.

### 2.4. Method Application

**Figure 4:**
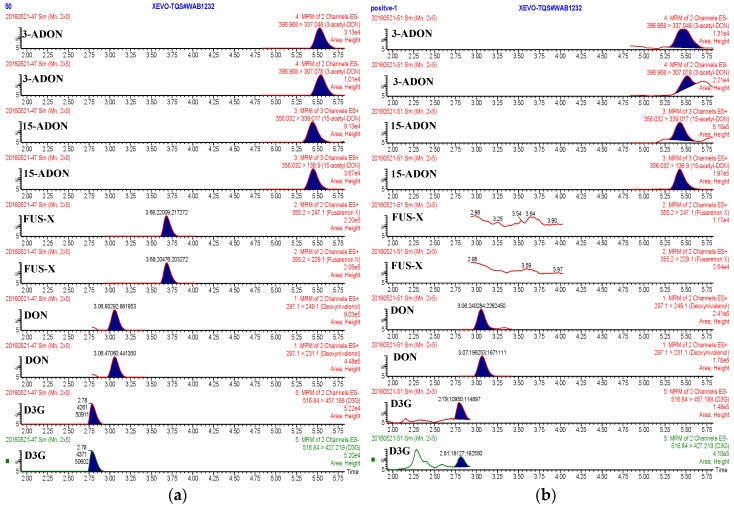
MRM chromatograms of five targeted mycotoxins in the standard solutions (**a**) and in a contaminated concentrated feed sample (**b**). The concentration of the mycotoxins in (**a**) is 50 μg/kg.

To further evaluate the method applicability, a total of 31 feed samples, including 11 formula feed, 12 premixed feed, and 8 concentrated feed samples were analyzed. The sample solutions with high concentration levels of the analytes were diluted with the blank matrices for fitting the linear range of the calibration curves. MRM chromatograms of the five mycotoxins in standard solutions and in a contaminated concentrated feed sample are shown in Figure 4. The results of multiple mycotoxins in different feed products are shown in Table 3.

Great variability in type and relative portions were demonstrated for the five mycotoxins in different feed samples. A total of 23 samples contained DON (incidence of 74.2%), the most prevalent mycotoxin, with the concentration range of 11.6–864.5 μg/kg, which were all lower than the maximum limit of 900 μg/kg in feeds set by European Commission [31], indicating low risks related to the intake of DON by animals in China. 3-ADON and 15-ADON were also found in feed samples with the incidences of 58.1% and 41.9%, respectively. FUS-X was only detected in two feed products, with the concentration in the range of 11.9–14.6 μg/kg. Surprisingly, D3G co-occurred with DON in most of the feed products with the incidence of 54.8% and the concentrations range of 2.1–34.8 μg/kg.

In total, 26 (incidence of 83.9%) feed samples were contaminated by different mycotoxins and all of them contained more than one analytes, indicating a high co-occurrence of multiple mycotoxins in feed samples. In view of the synergistic health risks related to the different type B trichothecenes and masked DON, in addition to the critical contamination situations found in the present study, continuous monitoring in various feed products by the developed UHPLC-MS/MS method is indeed indispensable.

## 3. Materials and Methods

### 3.1. Chemicals and Regents

Methanol and acetonitrile, both HPLC grade, were obtained from Merck (Darmstadt, Germany). Milli-Q quality water (Millipore, Billerica, MA, USA) was used throughout the whole analysis. All the other solvents were of analytical grade or HPLC grade. *GPD* HLB SPE cartridges were purchased from GuangPuDa Co. (Beijing, China). The standards of DON, 3-ADON, 15-ADON, and FUS-X were purchased from Sigma-Aldrich (St. Louis, MO, USA). D3G were obtained from Romer labs (Union, MO, USA).

### 3.2. Preparation of Standard Solutions

Accurately weighed solid portions of each standard were dissolved in acetonitrile to prepare 10 μg/mL of stock solution and stored at −20 °C in darkness. Each stock solution was diluted by acetonitrile/water containing 5 mmol/L ammonium acetate (20/80, *v*/*v*) to prepare a sequence of working solutions with the concentrations of 1, 2, 5, 10, 20, 50, 100, and 200 μg/kg, immediately before use.

### 3.3. Samples

A total of 31 feed samples were randomly collected from different regulated enterprises in China. According to the constituents and the processing crafts, the collected samples were included into three types: formula feed products (11 samples), concentrated feed products (eight samples) and premixed feed products (12 samples). All samples were grounded into powders by the high speed mill (Dingguang Mechanical Equipment Co., Ltd., Shanghai, China) to pass through a 2 mm sieve and stored at 4 °C until analysis.

### 3.4. Sample Preparation

Each sample (2.0 g) was macerated with 10 mL of acetonitrile/water solution (50:50, *v*/*v*) for 5 min, vortexed for 30 s, and then ultrasonicated for 40 min at 40 °C in ultrasonic bath (Shanghai Kedao Ultrasonic Instrument Co., Ltd., Shanghai, China). The extract was centrifuged at 4000 rpm for 5 min and an aliquot of 5 mL supernatant was collected and dried down with nitrogen gas at 45 °C. The residues were re-dissolved in 5 mL water, and passed through the *GPD* HLB cartridges, which were pre-conditioned with 3 mL methanol and 3mL water, at a flow rate of 1–2 drops/s. Then, the cartridges were washed with 5 mL water. Finally, all targeted analytes were eluted with 5 mL methanol, dried down with nitrogen gas at 45 °C, and re-dissolved in 1 mL acetonitrile/water containing 5 mmol/L ammonium acetate (20/80, *v*/*v*). The solution was passed through a 0.22 μm nylon filter (Pall, Port Washington, NY, USA) before UHPLC-MS/MS analysis.

### 3.5. UHPLC-MS/MS Analysis

A Waters ACQUITY UHPLC system coupled with a Waters XEVO TQ-S mass spectrometer (Waters) was used for simultaneous determination of DON, 3-ADON, 15-ADON, FUS-X and D3G in animal feed products. Chromatographic separation was performed on an ACQUITY UPLC^®^HSS T3 (2.1 × 150 mm, 1.8 μm; Waters) column with the mobile phase consist of methanol (A) and water containing 1 mmol/L ammonium acetate (B). Linear gradient elution program was designed as follows: initial 10% (A), 1 min 40% (A), 6 min 45% (A), 6.7 min 100% (A), 7.7 min 100% (A), 8 min 10% (A) and hold on for a further 2 min for re-equilibration, giving a total run time of 10 min. The flow rate was 0.4 mL/min and the injection volume was 3 μL. The column temperature and sample temperature were maintained at 40 °C and 5 °C, respectively. For MS/MS analysis, the electrospray ionization source was operated in both positive mode (ESI^+^) and negative mode (ESI^−^) with the parameters set as follows: interface voltages of capillary, 2.5 kV; voltages of cone, 25 V (ESI^+^) and 20 V (ESI^−^), desolvation temperature, 500 °C; source temperature, 150 °C. Nebulizing gas and desolvation gas flow rates were set as 7.0 bar and 1000 L/h, respectively. Multiple reaction monitoring (MRM) mode was developed for quantification of targeted mycotoxin (Table 4). MassLynx v4.1 and Targetlynx (Waters) was used for statistical analysis.

### 3.6. Method Validation

The standard solutions of DON, 3-ADON, 15-ADON, FUS-X, and D3G with the concentrations of 1, 2, 5, 10, 20, 50, 100, and 200 μg/kg were prepared in acetonitrile/water containing 5 mmol/L ammonium acetate (20/80, *v*/*v*) solution and blank feed matrices, respectively. Calibration curves were created by plotting the responses *versus* the concentrations of each analyte. When using a low resolution mass spectrometry, the two MRM transitions should produce signals distinguishable from the background ion current for ascertaining the target compound presence [32,33,34]. According to that, LOD and LOQ, which were used for evaluation of the sensitivity of the established method, were designed as the concentrations of the relative analyte that could provide a signal-to-noise ratio(S/N) of 3/1 for the second intense transition and 10/1 for the most intense transition, respectively. They were determined by serial dilution of mycotoxins spiked matrix with the blank matrix. Matrix effects were assessed by determining signal suppression/enhancement (SSE) values according to the following formula [34,35,36]:

SSE(%) = 100 × slope of matrix matched calibration curves/slope of standard calibration curves(1)

Recovery, intra- and inter-day precision tests were performed in quintuplicate using blank feed samples spiked with low, intermediate and high concentration levels (10, 50, and 200 μg/kg) of each mycotoxin. Recovery was calculated by comparing the measured concentration with the spiked concentration of each analyte. The relative standard deviations (RSDs) determined in the same day were used for evaluation of the intra-day precision and the values in five consecutive days were used for inter-day precision.

## 4. Conclusions

An efficient UHPLC-MS/MS method was developed for simultaneous determination of four type B trichothecenes and masked DON (D3G) in various feed products. An improved sample clean-up approach based on commercially available HLB cartridges was established for the first time making the analytical method more reliable and sensitive compared to the previous studies. After validation by determining the linearity, sensitivity, recovery, precision, and further successful application in real feed samples detection, the established method was proven to be a powerful tool for accurate quantification of these mycotoxins in formula feed, concentrated feed, and premixed feed products. Considering the highly toxic characteristics of all these mycotoxins alongside their critical incidence and contamination levels, it is recommended to include the mycotoxins not only the original forms but also their masked forms in routine monitoring and control programs for various feed products.

## Figures and Tables

**Table 1:** Calibration curves and sensitivities of five targeted mycotoxins in neat solvents and various feed products, respectively.

Matrix	Mycotoxins	Linear Range (μg/kg)	Slope	Intercept	*R*^2^	LOD ^a^	LOQ ^b^
			(*X* ± SD)	(*X* ± SD)		(μg/kg)	(μg/kg)
Neat solvents	DON	1–200	519 ± 57	3153 ± 125	0.999		
	3-ADON	2–200	90 ± 7	−108 ± 14	0.999		
	15-ADON	1–200	238 ± 14	140 ± 13	0.997		
	FUS-X	1–200	447 ± 25	−82 ± 7	0.999		
	D3G	1–200	98 ± 8	45 ± 4	0.999		
Formula feed	DON	1–200	485 ± 18	1197 ± 57	0.999	0.08	0.10
	3-ADON	5–200	70 ± 7	26 ± 4	0.996	2.09	4.17
	15-ADON	2–200	152 ± 13	972 ± 48	0.991	0.57	1.21
	FUS-X	2–200	335 ± 14	294 ± 11	0.998	0.44	1.93
	D3G	1–200	75 ± 4	729 ± 54	0.990	0.46	0.93
Concentrated feed	DON	1–200	414 ± 19	45368 ± 234	0.995	0.23	0.52
	3-ADON	5–200	59 ± 8	357 ± 24	0.997	2.31	4.85
	15-ADON	2–200	100 ± 9	3472 ± 98	0.995	0.98	1.86
	FUS-X	2–200	154 ± 8	272 ± 18	0.999	0.68	1.57
	D3G	1–200	70 ± 5	438 ± 21	0.993	0.42	0.98
Premixed feed	DON	1–200	462 ± 17	3404 ± 68	0.990	0.12	0.24
	3-ADON	5–200	84 ± 4	−108 ± 5	0.999	1.32	2.98
	15-ADON	2–200	202 ± 18	3020 ± 152	0.997	0.74	1.86
	FUS-X	2–200	381 ± 21	−440 ± 26	0.998	0.58	1.24
	D3G	1–200	89 ± 5	36 ± 8	0.994	0.29	0.7

**^a^** Limit of detection (S/N = 3, transition: 297.1 > 231.1 for DON, 396.9 > 307.1 for 3-ADON, 356.1 > 136.9 for 15-ADON, 355.2 > 229.1 for FUS-X and 517.2 > 427.1 for D3G). ^b^ Limit of quantitation (S/N = 10, transition: 297.1 > 249.1 for DON, 396.9 > 337.1 for 3-ADON, 356.1 > 339.1 for 15-ADON, 355.2 > 247.1 for FUS-X and 517.2 > 457.1 for D3G).

**Table 2:** Recovery, intra- and inter-day precision of five mycotoxins in various feed products (%, *n* = 5).

Mycotoxins	Spiked Levels (μg/kg)	Formula Feed			Concentrated Feed			Premixed Feed		
		Recovery	Intra-RSD	Inter-RSD	Recovery	Intra-RSD	Inter-RSD	Recovery	Intra-RSD	Inter-RSD
		(*X* ± SD)			(*X* ± SD)			(*X* ± SD)		
DON	10	102.3 ± 11.8	11.6	11.9	91.9 ± 7.8	8.4	7.9	95.6 ± 2.7	2.8	10.2
	50	95.7 ± 3.9	4.8	2.6	78.0 ± 5.5	7.1	7.7	98.5 ± 5.3	5.4	10.7
	200	91.5 ± 5.0	5.5	6.1	94 ± 1.9	2.1	6.1	94.2 ± 10.8	11.5	13.2
3-ADON	10	89.5 ± 10.1	3.6	11.3	92.1 ± 8.7	11.5	9.5	86.4 ± 9.6	10.3	11.2
	50	92.3 ± 9.6	11.5	10.4	93.0 ± 10.1	3.5	10.9	85.6 ± 8.5	8.5	9.9
	200	92.9 ± 8.1	6.1	8.7	88.9 ± 9.9	5.9	10.2	88.3 ± 9.1	7.2	10.3
15-ADON	10	96.1 ± 9.5	2.7	9.8	94.7 ± 8.6	8.4	9.1	89.5 ± 9.9	11.9	11.1
	50	90.5 ± 7.7	8.6	8.5	94.4 ± 9.8	4.8	10.4	84.1 ± 5.7	2.9	6.8
	200	92.6 ± 9.7	11.2	10.5	88.0 ± 8.9	9.0	10.1	96.2 ± 10.7	9.6	9.9
FUS-X	10	95.1 ± 7.3	7.6	9.7	95.1 ± 12.7	13.3	8.9	82.4 ± 4.8	5.9	11.8
	50	101.1 ± 7.5	7.4	7.5	81.0 ± 6.6	8.2	11.5	100.1 ± 5.3	5.3	11.5
	200	97.6 ± 1.1	1.1	4.2	93.6 ± 3.5	3.7	10.7	78.5 ± 6.5	8.3	11.6
D3G	10	99.1 ± 10.5	10.6	11.2	101.8 ± 13.5	13.2	8.4	80.1 ± 4.6	5.7	12.9
	50	85.7 ± 4.0	4.6	5.6	82.5 ± 11.2	13.5	14.9	108.1 ± 8.5	7.8	14.6
	200	89.2 ± 1.9	2.1	4.1	83.9 ± 9.9	11.7	13.4	79.3 ± 2.3	2.9	13.2

**Table 3:** Occurrence of D3G and four major type B trichothecenes in formula feed, concentrated feed, and premixed feed samples.

Mycotoxin	Formula Feed		Concentrated Feed		Premixed Feed	
	Positive/Total Samples	Range (μg/kg)	Positive/Total Samples	Range (μg/kg)	Positive/Total Samples	Range (μg/kg)
**DON**	9/11	47.1–864.5	6/8	11.6–277.6	8/12	97.4–776.3
**3-ADON**	8/11	5.1–221.8	5/8	5.6–56.4	5/12	26.5–135.1
**15-ADON**	6/11	5.0–350.4	5/8	5.7–160.2	2/12	99.5–332.8
**FUS-X**	0/11	ND	2/8	11.9–14.6	0/12	ND
**D3G**	8/11	2.1–21.6	3/8	3.5–34.8	6/12	2.1–30.1

ND: Not Detected.

**Table 4:** MS/MS parameters of the five mycotoxins.

Mycotoxin	Retention Time (min)	Precursor Ion (*m*/*z*)	Products Ion (*m*/*z*)	Collision Energy (eV)
DON	3.1	297.1 [M + H]^+^	249.1 *	10
			231.1	13
3-ADON	5.6	396.9 [M + CH3COO]^−^	337.1 *	14
			307.1	8
15-ADON	5.5	356.1 [M + NH4]^+^	339.1 *	12
			136.9	6
FUS-X	3.8	355.2 [M + H]^+^	247.1 *	13
			229.1	15
D3G	2.8	517.2 [M − H]^−^	457.1 *	14
			427.1	22

* Quantitative ion.

## Abbreviations

The following abbreviations are used in this manuscript:

DON	deoxynivalenol
3-ADON	3-acetyldeoxynivalenol
15-ADON	15-acetyldeoxynivalenol
FUS-X	fusarenon X
D3G	deoxynivalenol-3-glucoside
UHPLC-MS/MS	ultra-high performance liquid chromatography-tandem mass spectrometry
TLC	thin-layer chromatography
ELISA	enzyme-linked immune-sorbent assay
HPLC	high-performance liquid chromatography
ESI	electrospray ionization
APCI	atmospheric pressure chemical ionization
LLE	liquid liquid extraction
DSPE	dispersive solid-phase extraction
SPE	solid phase extraction
LOD	limit of detection
LOQ	limit of quantification
MRM	multiple reaction monitoring
S/N	signal-to-noise ratio
SSE	signal suppression/enhancement
RSD	relative standard deviation
